# Ponatinib Improved the Prognosis of Philadelphia Chromosome-Positive Acute Lymphoblastic Leukemia: A Japanese Single-Center Cohort Study

**DOI:** 10.7759/cureus.50416

**Published:** 2023-12-12

**Authors:** Nagi Tozawa, Takaya Yamashita, Miho Nara, Yuki Fujioka, Sho Ikeda, Takahiro Kobayashi, Isuzu Kobayashi, Akihiro Kitadate, Yoshihiro Kameoka, Naoto Takahashi

**Affiliations:** 1 Department of Hematology, Akita University Hospital, Akita, JPN

**Keywords:** treatment-free remission, philadelphia chromosome, cd20, molecular complete remission, maintenance therapy, allogeneic hematopoietic stem cell transplantation, ponatinib, tyrosine kinase inhibitor, acute lymphocytic leukemia

## Abstract

Introduction

The overall survival (OS) of Philadelphia chromosome-positive acute lymphoblastic leukemia (Ph+ALL) has improved with the combination of tyrosine kinase inhibitor (TKI) with intensive chemotherapy. In recent years, there has been increased interest in the possibility of long-term survival without allogeneic hematopoietic stem cell transplantation (HSCT) or maintenance therapy. The aim of this study was to determine the effectiveness of treatment and the resultant outcomes in Ph+ALL patients using real-world data.

Methods

We performed a single-center retrospective analysis utilizing Akita University Hospital data (Akita, Japan) from November 2000 to June 2023 to evaluate the outcomes of TKI with intensive chemotherapy for Ph+ALL.

Results

Twenty-three patients with Ph+ALL were treated with intensive chemotherapy combined with TKI, including six imatinib, four dasatinib, and 13 ponatinib. The median patient age was 53 years (range; 28-67). Eighteen patients (78%) achieved complete molecular remission (CMR) within three months. HSCT was performed in 16 patients (70%), all of whom did not receive post-transplant TKI maintenance therapy. Six of the seven patients who did not undergo HSCT received maintenance therapy with ponatinib after intensive chemotherapy. The three-year OS was 81%. Ponatinib treatment resulted in a much higher OS rate than imatinib/dasatinib (100% vs. 60%; *P*=0.011). CMR within three months was identified as a prognostic factor for molecular relapse-free survival (hazard ratio (HR)=0.22; *P*=0.027). CD20 positivity was identified as a risk factor for hematological relapse (HR=5.2, *P*=0.032).

Conclusion

Even in a single-center cohort study, ponatinib, as a combination TKI with intensive chemotherapy or maintenance therapy, may improve the prognosis of Ph+ALL. Patients with CMR within three months might not necessarily need to receive HSCT, but a subsequent treatment-free status could have been achieved only by HSCT. Furthermore, CD20 positivity may be a useful biomarker for future treatment decisions in patients with Ph+ALL.

## Introduction

Philadelphia chromosome-positive acute lymphoblastic leukemia (Ph+ALL) is a hematological malignancy caused by the BCR::ABL1 fusion gene resulting from a t(9;22) chromosomal translocation that results in the acquisition of constant ABL1 tyrosine kinase activity, promoting leukemic cell survival and proliferation. Ph+ALL accounts for 20-30% of adult ALL cases, and its frequency increases to 50% in those aged >60 years [1]. In the past, the Philadelphia chromosome was an independent risk factor for survival among adult patients with ALL with a probability of long-term survival <10%; however, with the combination of tyrosine kinase inhibitor (TKI) imatinib and chemotherapy regimens, the five-year overall survival (OS) rate of Ph+ALL has dramatically improved to over 50% [2-6].

Among chemotherapy regimens combined with imatinib, hyperfractionated cyclophosphamide, vincristine, adriamycin, and dexamethasone/high-dose methotrexate and cytarabine (hyper-CVAD/MA) has been reported to be highly effective for Ph+ALL by the MD Anderson Cancer Center (MDACC) [6]. They also reported a phase 2 trial in Ph+ALL in which imatinib was switched to the second-generation TKI dasatinib or third-generation TKI ponatinib to overcome imatinib resistance [7-9]. In particular, ponatinib has been shown to achieve higher remission rates and improve prognosis than other TKIs [10]. Even in the TKI era, allogeneic hematopoietic stem cell transplantation (HSCT) in the first remission remains the standard of care for Ph+ALL according to the European Group for Blood and Marrow Transplantation (EBMT) guidelines [11] or Japanese Society of Hematology guidelines [12]. By contrast, the National Comprehensive Cancer Network (NCCN) guidelines recommend HSCT as a treatment option, even for patients who achieve measurable residual disease (MRD)-negative first remission [13].

Although the OS of patients with Ph+ALL has improved with the combination of TKI with intensive chemotherapy, the need for HSCT or maintenance therapy has not yet been determined. Here, we conducted a retrospective cohort study based on the clinical information of Ph+ALL cases treated at Akita University Hospital, Akita, Japan, since the introduction of imatinib, comparing treatment efficacy and outcome according to patient background and treatment. Prognostic factors, the need for HSCT, and maintenance therapy after HSCT are discussed based on our recent clinical data.

## Materials and methods

Patients

This was a single-center, retrospective cohort study conducted at Akita University Hospital, Akita, Japan. Patients aged ≥20 years were newly diagnosed with Ph+ALL and received intensive chemotherapy with TKI between November 2000 and June 2023. To reduce the impact of patient background differences, this study excluded patients who were unable to receive intensive chemotherapy due to older age, comorbidities, or poor performance status. Clinical data obtained from medical records were retrospectively analyzed. This study was conducted in accordance with the Declaration of Helsinki and was reviewed and approved by the Ethics Committee of Akita University (approval no. 2962). Because this was a retrospective study that did not involve clinical specimens, consent was obtained from eligible patients on an opt-out basis.

Outcomes

The start date was set as the date the TKI therapy was initiated. The primary objective was to assess the rate of complete molecular remission (CMR) achievement within three months in patients receiving intensive chemotherapy with TKIs. The secondary objectives were to determine three-year OS and relapse-free survival (RFS) in the study participants. The study also aimed to compare OS and RFS with and without HSCT and according to the type of TKI. Hematological complete remission was defined as encompassing the complete remission (CR), CR with partial hematologic recovery, and CR with incomplete hematologic recovery criteria detailed in the NCCN guidelines, while hematological relapse was defined as the loss of these conditions [13]. CMR was defined as a reduction in BCR::ABL1 transcript levels by real-time PCR below the assay’s sensitivity on a bone marrow sample. The assay has a detection limit of 50 copies/µgRNA. Molecular relapse was defined as an increase in the BCR::ABL1 transcript level over time above the sensitivity of detection. OS was defined as the period from TKI initiation to the date of death from any cause or the date of last confirmed survival. Hematological relapse-free survival (H-RFS) and molecular relapse-free survival (M-RFS) were defined as the time from the date of TKI initiation to the date of hematological relapse or molecular relapse or death from any cause or the last confirmed survival, whichever comes first.

Statistical analysis

The data cutoff date was June 30, 2023. The Kaplan-Meier method was used for survival analysis. For univariate analysis, a Cox proportional hazards model was used. Fisher's exact test was used for nominal variables, and the t-test or Mann-Whitney U test was used for continuous variables. EZR statistical software (CRAN (The Comprehensive R Archive Network) as "RcmdrPlugin.EZR" package) was used for all statistical analyses [14].

## Results

Twenty-three patients with Ph+ALL were treated with intensive chemotherapy, such as hyper-CVAD/MA or Japan Adult Leukemia Study Group (JALSG) ALL regimens combined with TKI [2,10]. The patient backgrounds are shown in Table 1. The median age was 53 years (range: 28-67), and two patients (9%) were 65 years or older. Minor BCR::ABL1 (p190), major BCR::ABL1 (P210), and additional chromosomal abnormalities were found in 11 (49%), 11 (49%), and 16 patients (70%), respectively. The first TKI administered with intensive chemotherapy were imatinib, dasatinib, and ponatinib in six (26%), four (17%), and 13 patients (57%), respectively.

**Table 1:** Patient characteristics. Values at P < 0.05 were considered statistically significant. WBC, white blood cell; Hb, hemoglobin; Plt, platelet; LD, lactate dehydrogenase; BM, bone marrow; CNS, central nervous system; TKI, tyrosine kinase inhibitor; CMR, complete molecular remission; HSCT, allogeneic hematopoietic stem cell transplantation; PBSC, peripheral blood stem cell

	Total (n=23)	IM/DAS (n=10)	PON (n=13)	P-value
Gender, n (%)				1
Male	10 (43)	4 (40)	6 (46)	
Female	13 (57)	6 (60)	7 (54)	
Age at diagnosis, median (range)	53 (28-67)	46 (31-60)	57 (28-67)	0.13
20-64, n (%)	21 (91)	10 (100)	11 (85)	
≧65, n (%)	2 (9)	0 (0)	2 (15)	
Labo data at diagnosis, median (range)				
WBC (×10^3^/μL)	22.6 (2.2-679.2)	22.5 (1.6-524.8)	22.6 (3.6-679.2)	0.47
Hb (g/dL)	11.5 (4.3-14.7)	8.3 (4.3-18.5)	12.7 (5.5-14.7)	0.079
Plt (×10^3^/μL)	63 (3-306)	54 (3-171)	70 (3-306)	0.66
LD (IU/L)	847 (242-7,162)	715 (291-1,938)	992 (242-7,162)	0.41
BM blast (%)	89.8 (21.6-97.2)	94.9 (60-97.2)	83.8 (21.6-96.4)	0.007
BCR::ABL1 mRNA (copy/μgRNA)	2.2×10^5^ (1.9×10^2^-9.5×10^5^)	2.0×10^4^ (1.9×10^2^-9.5×10^5^)	2.2×10^5^ (4.4×10^4^-9.5×10^5^)	0.22
CD20 >20%, n (%)	6 (26)	4 (40)	2 (15)	0.34
ECOG performance status, n (%)				1
0-1	22 (96)	10 (100)	12 (92)	
≧2	1 (4)	0 (0)	1 (8)	
Cytogenetic abnormalities, n (%)				0.65
t(9;22) isolated	6 (26)	2 (20)	4 (31)	
t(9;22) with additional chromosomal abnormalities	16 (70)	8 (80)	8 (62)	
Unknown	1 (4)	0 (0)	1 (8)	
Isoform, n (%)				1
p190(minor)	11 (49)	5 (50)	6 (46)	
p210(major)	11 (49)	4 (40)	7 (54)	
Unknown	1 (4)	1 (10)	0 (0)	
CNS involvement, n (%)	3 (13)	2 (20)	1 (8)	0.56
TKI, n (%)				
Imatinib	6 (26)	6 (60)	0 (0)	
Dasatinib	4 (17)	4 (40)	0 (0)	
Ponatinib	13 (57)	0 (0)	13 (100)	
CMR within 3 month, n (%)	18 (78)	6 (60)	12 (92)	0.13
HSCT, n (%)	16 (70)	9 (90)	7 (54)	0.089
PBSC of matched related donor	4 (25)	4 (44)	0 (0)	
BM of matched unrelated donor	4 (25)	3 (33)	1 (14)	
BM of mismatched unrelated donor	5 (31)	1 (11)	4 (57)	
Cord blood	3 (19)	1 (11)	2 (29)	
Maintenance therapy				
Post HSCT, n (%)	0 (0)	0 (0)	0 (0)	1
Without HSCT, n (%)	6 (85)	0 (0)	6 (100)	0.008
ABL mutation, n (%)	1 (4)	1 (10)	0 (0)	0.44

**Figure 1:**
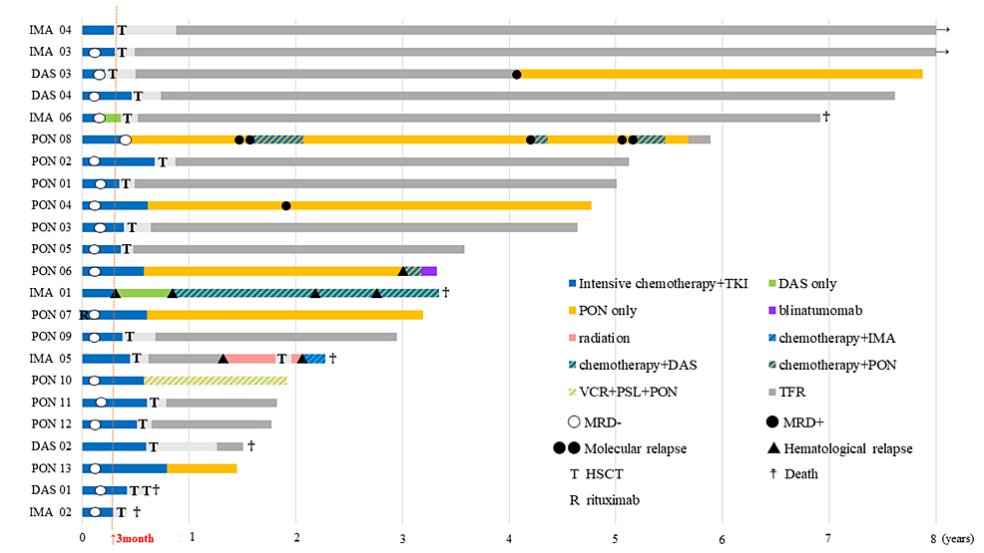
Clinical course of all 23 patients. P values were calculated using the log-rank test. TKI, tyrosine kinase inhibitor; IMA, imatinib; DAS, dasatinib; PON, ponatinib; VCR, vincristine; PSL, prednisolone; TFR, treatment free remission; MRD, measurable residual disease; HSCT, allo stem cell transplantation

The clinical courses of all 23 patients are shown in a swimmer plot (Figure 1). The median follow-up for surviving patients was 136 months (range 92-195 months) in the imatinib/dasatinib group and 40 months (range 17-71 months) in the ponatinib group. Twenty-one (91%) patients achieved hematological complete remission. Eighteen (78%) patients achieved CMR within three months. HSCT was performed in 16 patients (70%), none of whom received post-transplant TKI maintenance therapy. Among the seven patients who did not receive HSCT, all received TKI as maintenance therapy after intensive chemotherapy (100%). One patient (4%) in the imatinib/dasatinib group had a point mutation with T315I during the clinical course. Central nervous system (CNS) involvement was observed in three patients (13%). Six patients (60%) treated with imatinib/dasatinib died of transplant-related complications without relapse in three patients (IMA02, DAS01, and DAS02), relapse/progressive disease in two (IMA01 and IMA05), and progression of transplantation-related interstitial pneumonia in one patient (IMA06). Despite the differences in the observation periods, none of the patients treated with ponatinib died before the cut-off date.

**Figure 2:**
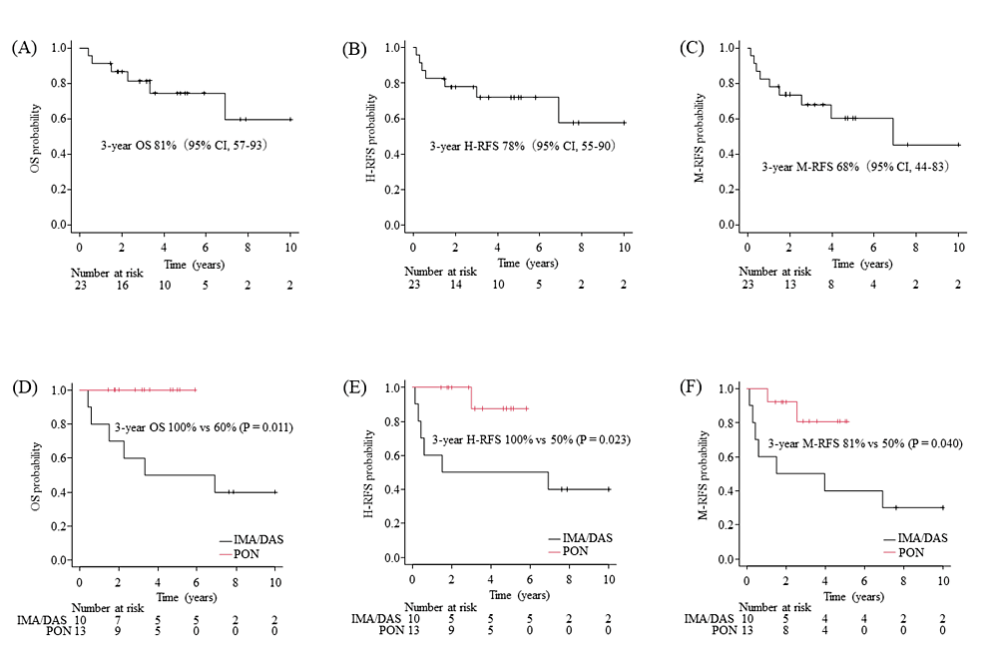
Kaplan-Meier plot of (A) overall survival, (B) hematological relapse-free survival, and (C) molecular relapse-free survival for all patients (n=23). Kaplan-Meier plot of (D) overall survival, (E) hematological relapse-free survival, and (F) molecular relapse-free survival stratified by TKIs, imatinib/dasatinib, or ponatinib. P values were calculated using the log-rank test. IMA, imatinib; DAS, dasatinib; PON, ponatinib; 95% CI, 95% confidence interval

The three-year OS, H-RFS, and M-RFS rates for all 23 patients were 81%, 78%, and 68%, respectively (Figure 2A-2C). The OS, H-RFS, and M-RFS stratified by TKIs, imatinib/dasatinib, or ponatinib are shown as Kaplan-Meier curves (Figure 2D-F). The log-rank test showed a statistically significant long-term survival advantage for the ponatinib group compared to the imatinib/dasatinib group.

In a univariate analysis (Table 2), the hazard ratio (HR) for H-RFS was significantly higher in patients with ≥20% of CD20 expression in leukemic cells compared to CD20 negative cases (HR=5.2, P=0.032). The HR for M-RFS was significantly lower in patients who achieved CMR within three months (HR=0.22, P=0.027).

**Table 2:** Univariate analysis for the outcomes in patients treated with TKI (n=23). Values at P < 0.05 were considered statistically significant. ^#^ Univariate analyses were performed on these items as continuous variables. * Could not be analyzed because the proportional hazards property was not established. PON, ponatinib; IMA, imatinib; DAS, dasatinib; OS, overall survival; H-RFS, hematological relapse free survival; M-RFS, molecular relapse free survival; HR, hazard ratio; 95%CI, 95% confidence interval; BM, bone marrow; ACAs, additional chromosomal changes;  TKI, tyrosine kinase inhibitor;  CMR, complete molecular remission; HSCT, allogeneic hematopoietic stem cell transplantation

	OS				H-RFS				M-RFS			
	HR (95%CI)			P	HR (95%CI)			P	HR (95%CI)			P
Gender, Male	1.55 (0.31, 7.84)			0.59	1.08 (0.24, 4.84)			0.92	1.19 (0.31, 4.50)			0.8
Age^#^	0.97 (0.90, 1.05)			0.44	0.98 (0.92, 1.06)			0.66	1.02 (0.95, 1.09)			0.62
WBC^#^	0.57 (0.16, 2.05)			0.39	0.53 (0.15, 1.83)			0.32	0.60 (0.21, 1.76)			0.35
BM blast %	1.18 (0.84, 1.66)			0.33	1.11 (0.91, 1.36)			0.30	1.07 (0.97, 1.18)			0.20
CD20, >20%	4.37 (0.85, 22.42)			0.077	5.21 (1.16, 23.48)			0.031	3.33 (0.88, 12.62)			0.076
mRNA^#^	0.65 (0.27, 1.55)			0.33	0.76 (0.34, 1.71)			0.51	0.89 (0.44, 1.80)			0.73
ACAs, yes	0.82 (0.15-4.50)			0.82	0.88 (0.17, 4.53)			0.87	1.41 (0.29, 6.80)			0.67
Isoform, minor	0.40 (0.07, 2.25)			0.30	0.69 (0.15, 3.18)			0.63	0.77 (0.21, 2.91)			0.70
TKI used in induction regimen, PON	NA*				0.12 (0.01, 1.05)			0.056	0.24 (0.04, 1.07)			0.061
CMR within 3 months, yes	0.30 (0.06, 1.50)			0.14	0.28 (0.06, 1.25)			0.094	0.22 (0.06, 0.85)			0.027
HSCT, yes	2.80 (0.29, 26.71)			0.37	1.72 (0.22, 13.55)			0.60	1.55 (0.19, 12.78)			0.68

**Figure 3:**
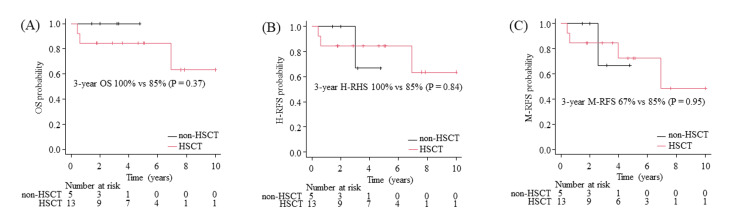
Kaplan-Meier plot of (A) overall survival, (B) hematological relapse-free survival, and (C) molecular relapse-free survival for patients stratified by HSCT for patients who achieved molecular complete remission within three months. P values were calculated using the log-rank test. HSCT, hematopoietic stem cell transplantation

To examine the need for HSCT, 18 patients with confirmed CMR within three months of TKI treatment combined with intensive chemotherapy were evaluated. Of the 18 patients, 13 underwent HSCT, and the other five did not. All five patients were treated with ponatinib as the first TKI combined with intensive chemotherapy. The OS, H-RFS, and M-RFS stratified by HSCT are shown as Kaplan-Meier curves (Figure 3A-3C). There were no statistically significant differences in the long-term survival advantage between the HSCT group (n=13) and the non-HSCT group (n=5) by the log-rank test.

In addition, among patients who underwent HSCT (n=16), graft-versus-host disease (GVHD)-free, relapse-free survival (GRFS) was achieved in eight patients (50%). In the ponatinib group, GRFS was achieved in five patients (71%). Notably, post-transplant TFR, that is, maintenance-free status, was achieved in all patients who underwent HSCT, except for two patients who died early after transplantation due to transplant-related complications. On the other hand, among patients without HSCT (n=7), six received maintenance by ponatinib for 2.6 years (0.7-4.3 years).

## Discussion

As our institution is a regional transplant center, the patients analyzed in this single-center cohort study were relatively young and eligible for transplantation and received intensive chemotherapy combined with TKI. The three-year OS of all patients was approximately 81%, comparable to previously reported results [2-9].

The determinant with the most pronounced impact on the long-term prognosis within our cohort was identified as the specific TKI employed. Long-term prognosis was stratified into the imatinib/dasatinib and ponatinib groups and compared using Kaplan-Meier curves, showing the superiority of the ponatinib group. Of the 13 patients in the ponatinib group, 12 (92%) attained CMR within three months, in contrast to six out of 10 (60%) in the imatinib/dasatinib group. While not a statistically significant difference between the two groups, ponatinib may contribute to a better long-term prognosis than imatinib/dasatinib. Ponatinib is a TKI with the strongest inhibition of ABL kinase activity, and although only one case of T315I mutation was observed in this cohort, ponatinib is known to show inhibitory activity against ABL1 mutations, including T315I, to which imatinib/dasatinib is resistant [15]. This pharmacological feature of ponatinib may be one of the reasons why the CMR rate at three months was higher in the ponatinib group than in the imatinib/dasatinib group in this study, as well as in previous reports [10,16]. Furthermore, CMR within three months was shown in the univariate analysis as a predictor of M-RFS (HR=0.22, P=0.027). It has been reported that the prognosis of patients who achieved CMR within three months did not change with or without transplantation [16,17]. In addition, a multicenter retrospective study from the United States reported that in patients with Ph+ALL who achieved CMR within 90 days of remission induction therapy, including TKIs, OS did not change with or without HSCT in the first remission [18]. These reports demonstrate the existence of a group of patients who can survive for long periods without the need for HSCT. Therefore, it may be possible to avoid HSCT in cases with good treatment response. In our cohort, there was no significant difference in long-term survival between the groups that did and did not undergo HSCT in patients who achieved CMR within three months. Out of the 13 patients observed in the ponatinib group, seven (54%) underwent HSCT, while nine (90%) of the 10 patients in the imatinib/dasatinib group had the procedure. This indicates a greater tendency for patients in the ponatinib group to avoid HSCT. Therefore, none of the patients in the ponatinib group had transplant-related deaths compared to three in the imatinib/dasatinib group, which may be the most significant reason for the lower long-term prognosis in the imatinib/dasatinib group than in the ponatinib group.

One advantage of HSCT is that posttransplant maintenance therapy is not necessarily required, allowing for treatment-free follow-up. In our cohort, none of the patients who underwent HSCT received post-transplant maintenance therapy. All 13 patients who achieved CMR within three months and underwent HSCT achieved TFR without post-transplant maintenance therapy, except for two patients who died early after transplantation due to transplant-related complications. By contrast, the PONALFIL trial used post-transplant maintenance therapy as a preemptive dose, and 18 of 26 patients (69%) achieved post-transplant TFR [19]. Although several reports support the benefit of TKI maintenance therapy after HSCT in preventing recurrence [20-22], there are also reports of early discontinuation after initiation owing to TKI-related adverse events and problems in controlling post-transplant complications [23]. In a phase 2 PACE (Ponatinib Ph+ ALL and CML Evaluation) study, arterial occlusive events (AOEs) occurred in more than 25% of patients, as reported by the investigators [24]. This cannot be ignored as a late side effect. In HSCT cases without CMR, post-transplant maintenance therapy with preemptive administration for a defined period may prevent recurrence, whereas HSCT cases with early CMR may not require post-transplant maintenance therapy.

If HSCT is not performed, the NCCN guidelines recommend ponatinib maintenance therapy [13]. In our cohort, six of the seven patients who did not undergo HSCT received maintenance therapy with ponatinib; however, it is unclear how long ponatinib is needed. The hyper-CVAD/MA plus ponatinib regimen reported by the MDACC also includes ponatinib therapy for up to eight years when all intensive chemotherapy (approximately eight months) and two years of maintenance therapy with POMP (PSL, vincristine, and 6MP) plus more than five years of single-agent ponatinib maintenance therapy are added [9]. To reduce AOEs with ponatinib, the dose of ponatinib should be reduced from 45 to 30 mg/15 mg depending on the molecular response, and the duration of ponatinib administration should be as short as possible. Regarding the indication for transplantation in patients with Ph+ALL who have achieved CMR within three months, we should consider whether or be performed by weighing the risk of transplant-related mortality and AOEs as late side effects of long-term ponatinib administration.

Finally, this study suggests CD20 expression may be a poor prognostic factor for relapse-free survival in patients with Ph+ALL. In Ph-negative B-ALL, there are several previous reports where CD20 expression (>20%) increases relapse and worsens survival [25]. Based on these findings, a modified hyper-CVAD/MA therapy with rituximab is often administered. This therapy improved three-year OS compared to standard hyper-CVAD/MA in patients who were CD20-positive (75% vs. 47%, P=0.003) [26]. Since then, there have been reports of CD20 positivity as a favorable prognostic factor in Ph-negative B-ALL due to the addition of rituximab. A report from the United States showed that the HR for OS was 0.249 (P=0.02), and the HR for EFS was reduced to 0.147 (P<0.001) in Ph-negative B-ALL with CD20 positivity compared to CD20-negative cases [27]. However, the prognostic impact of CD20 positivity in Ph+ALL remains unclear. In this study, rituximab was not administered except in one case (PON07). CD20 positivity, identified as a factor involved in the RFS of Ph+ALL, may be overcome by the combination of rituximab, similar to CD20-positive Ph-negative B-ALL.

The first limitation of this study is the small number of patients. This was too small to perform a multivariate analysis; therefore, a univariate analysis was used for inference. Second, the retrospective design of this study and the lack of propensity score matching methods made it difficult to compare outcomes in different patient groups or historical backgrounds. The improved transplant outcomes may be due not only to the efficacy of ponatinib but also to advances in transplantation technology, including supportive care, which may suggest that HSCT can now be performed more safely. These results support the consideration of HSCT when indicated.

## Conclusions

We reviewed the Ph+ALL cases at Akita University Hospital. The prognosis of patients who receive intensive chemotherapy with TKI is relatively good. In particular, intensive chemotherapy with ponatinib can achieve early CMR and avoid HSCT and transplant-related mortality. The ponatinib group had a much higher survival rate than the imatinib/dasatinib group. Patients with CMR within three months may not have received HSCT; however, subsequent TFR can only be achieved by HSCT. CD20 positivity has been identified as a risk factor for relapse and may be a useful biomarker for future treatment decisions in patients with Ph+ALL.
